# Adolescents with perinatally acquired HIV — coming your way

**DOI:** 10.1186/1758-2652-13-S4-O36

**Published:** 2010-11-08

**Authors:** C Foster

**Affiliations:** 1 Imperial College Healthcare NHS Trust, London, UK

## 

In resourced settings with access to highly active antiretroviral therapy (HAART), perinatally acquired HIV-1 infection has become a chronic disease of childhood. Reduced mortality due to HAART, combined with high uptake of antenatal HIV testing and a marked reduction in rates of mother-to-child transmission has resulted in significant increases in the average age of paediatric cohorts in these regions and hence increasing numbers of children born with HIV are surviving to early adulthood and completing the process of transition from paediatric to adult services. Advances in antiretroviral therapy, reductions in pill burdens and drug side effects, new classes and new drugs within existing antiretroviral classes offer enormous benefits, although serious issues around adherence during adolescence continue. Questions persist around the optimal timing and sequencing of antiretroviral agents to maintain future treatment options, particularly with global recommendations for the earlier initiation of therapy across the paediatric age range. The longer term impact of exposure to HIV and antiretroviral therapy throughout childhood are becoming apparent, with growing concern over neurocognitive, cardiovascular, renal and bone health requiring further elucidation. Adolescents living with perinatally acquired HIV have additional psychosocial issues including the impact of HIV on other family members, roles as young carers, experience of parental and sibling bereavement, and live with a disease that is potentially transmissible to future sexual partners before they themselves have had sex. Increasing numbers of young women born themselves with HIV are becoming sexually active and having uninfected infants of their own and the long term outcomes for the next generation require monitoring. The benefits of HAART far outweigh the potential risks and the careful follow up of this early perinatal cohort as they progress through adulthood will hopefully aid the future management of the growing numbers of adolescents surviving worldwide as access to therapy improves.

